# Simultaneous Occurrence of Cytomegalovirus Colitis and Retinitis as the Initial Presentation of Human Immunodeficiency Virus Infection in a Patient With Zero CD4 Count

**DOI:** 10.7759/cureus.22455

**Published:** 2022-02-21

**Authors:** Adeeb Munshi, Hassan Almarhabi, Mohamed K Mujalled, Fatimah Alturkistani, Abdulhakeem Althaqafi

**Affiliations:** 1 Medicine/Infectious Diseases, King Abdulaziz Medical City, Jeddah, SAU; 2 Medicine/Infectious Diseases, King Abdullah International Medical Research Center, Jeddah, SAU; 3 College of Medicine, King Saud Bin Abdulaziz University for Health Sciences, Jeddah, SAU; 4 Internal Medicine, King Abdulaziz Medical City, Jeddah, SAU; 5 Internal Medicine, King Abdullah International Medical Research Center, Jeddah, SAU; 6 Anatomical Pathology, King Abdulaziz Medical City, Jeddah, SAU

**Keywords:** diarrhea, retinitis, colitis, cytomegalovirus (cmv), hiv

## Abstract

Cytomegalovirus (CMV) can be a reason for severe disease in immunocompromised patients, either via the reactivation of latent CMV infection or via the acquisition of primary CMV infection. Clinical syndromes that may be observed are retinitis, colitis, esophagitis, encephalitis, pneumonitis, hepatitis, uveitis, and graft rejection following transplantation. The simultaneous occurrence of CMV colitis and retinitis as the initial presentation of human immunodeficiency virus (HIV) infection is extremely rare. We present a case of a 42-year-old male, known to have type 2 diabetes, bronchial asthma, and allergic rhinitis, who presented to the emergency department (ED) with two weeks history of abdominal pain and watery diarrhea four to five times per day sometimes accompanied by small amounts of blood. He also had an on-and-off subjective fever for the last two months prior to presentation, loss of appetite, and weight loss of 13 kg over the last six months. Additionally, he had a history of travel outside Saudi Arabia and unprotected sex. HIV-1 serology (combination antigen/antibody immunoassay) was requested, which came out positive, with a viral load of 323141 copies/mL and decreased CD4+ T lymphocytes (0 cel/μL). CMV polymerase chain reaction (PCR) quantitative was detected and the CMV viral load was 694963 IU/mL. Given the patient's bloody diarrhea with positive CMV and HIV, the gastroenterology team was consulted and they decided to proceed with a colonoscopy and biopsy. The patient was diagnosed with CMV colitis based on the biopsy results. The patient also was found to have CMV retinitis based on ophthalmologic assessment. Multidisciplinary teams, including infectious diseases, gastroenterology, ophthalmology, and pathology, should cooperate to facilitate an accurate and fast diagnosis. Further complications can be prevented by early diagnosis andtreatment.

## Introduction

Cytomegalovirus (CMV) can be a reason for severe disease in immunocompromised patients, either via the reactivation of latent CMV infection or via the acquisition of primary CMV infection. CMV infection can be present in many forms. Clinical syndromes that may be observed are retinitis, colitis, esophagitis, encephalitis, pneumonitis, hepatitis, uveitis, and graft rejection following transplantation [1].

The human immunodeficiency virus (HIV) damages the immune system, as it targets CD4 cells [2]. CD4 cells are T-cells that protect the body against bacteria, viruses, and other invading germs. HIV grabs onto the surface of a cell, gets inside it, and becomes part of it. When infected CD4 cells are completely destroyed, they release more copies of HIV into the bloodstream [3]. These new streams of the virus find and attack more CD4 cells, and the cycle continues. This results in much fewer HIV-free working CD4 cells. HIV can destroy entire "families" of CD4 cells, and this allows opportunistic infections (OIs) to attack the body and causes serious illness [4].

CMV retinitis is commonly confused with HIV retinopathy; however, the latter fades and condenses over time while CMV retinitis progresses [5]. When left untreated, CMV retinitis can progress to retinal detachment with direct damage to the macula or optic nerve, resulting in permanent blindness [5]. Blindness caused by CMV retinitis is irreversible and can occur prior to complete retinal destruction, which was responsible for >90% of HIV-associated blindness in the pre-antiretroviral therapy (ART) era [6].

Colitis is the most common extra-ocular manifestation of CMV infection in HIV-infected patients [7]. CMV disease commonly appears in HIV-infected patients with CD4+ counts less than 50/mm3. CMV colitis should be considered in diarrheal patients with HIV infection even after initiation of ART [7].

In the case presented herein, CMV colitis and retinitis are presented as initial manifestations of HIV infection in a patient with a zero CD4 count.

## Case presentation

A 42-year-old male, known to have type 2 diabetes, bronchial asthma, and allergic rhinitis, presented to the Emergency Department (ED) with a two-week history of abdominal pain and watery diarrhea four to five times per day sometimes accompanied by small amounts of blood. He also had an on-and-off subjective fever for the last two months prior to presentation, loss of appetite, and weight loss of 13 kg over the last six months.

The patient was also complaining of progressive lower limb weakness and numbness bilaterally, for which he was started on pregabalin, however, the weakness was progressive to the extent that when the patient was seen, he was already bedridden for the last two weeks prior to his presentation. Additionally, he had a history of travel outside Saudi Arabia and unprotected sex. The patient also had a history of alcohol and cannabis use but denied a history of intravenous (IV) drug abuse, night sweat, contact with sick patients, cough, and shortness of breath. The further systematic review was unremarkable.

On examination in the ED, blood pressure was 106/68 mmHg, heart rate was 106 beats per minute, respiratory rate was 20 breaths per minute, temperature was 38°C, and oxygen saturation was 100% on ambient air. The patient has poor oral hygiene and normal cardiopulmonary and abdominal examination. Examination of the lymph nodes examination revealed multi-subcentimetric non-tender lymph nodes, one in the anterior and one in the posterior cervical lymph nodes group. Rectal examination showed severe anal pain, with multiple perianal ulcers and purulent discharge, and no blood or melena. Neurological examination revealed upper limb weakness bilaterally, as the power was 4/5 but intact sensation and reflux while lower limb showed hyporeflexia, hypotonia, and weakness bilaterally with power 3/5.

Laboratory investigations revealed a leukocyte count of 4.9 11.9 x10^9^/L and c-reactive protein (CRP) 70.6 mg/L. HIV-1 serology (combination antigen/antibody immunoassay) was requested, which came out positive, with a viral load of 323141 copies/mL and decreased CD4+ T lymphocytes (0 cel/μL). CMV polymerase chain reaction (PCR) quantitative was detected and CMV viral load was 694963 IU/mL (Table 1).

**Table 1:** Investigations of the patient upon admission INR: international normalized ratio; PT: prothrombin time; PTT: partial thromboplastin time; CMV: Cytomegalovirus; IgG: immunoglobulin G; PCR: polymerase chain reaction

Variable	Upon presentation
INR	1.0 (ref 0.8-12 sec)
PT	12 (ref 11-14sec)
PTT	43 (ref 26-41)
Platelet	212 (ref 150-450 x10^9^/L)
White-cell count	4.9 (ref 150-450 x10^9^/L)
Lymphocytes	0.88 (ref 1.5 - 4.0 x10^9^/L)
Neutrophils	3.53 (ref 2.00 - 7.50 x10^9^/L)
Haemoglobin	9.4 (ref 13 - 18 g/dL)
Lipase	87 (ref 8 - 78 U/L)
Amylase	101 (ref 31 - 118 U/L)
Lactic acid	2.14 (ref 0.7 - 2 mmol/L)
C-reactive protein	70.6 (ref 0 - 5 mg/L)
Creatinine	66 (ref 65 – 112 umol/liter)
Alanine aminotransferase	33 (ref 7 – 44 IU/liter)
Aspartate aminotransferase	77 (ref 5 – 34 IU/liter)
CMV IgG total antibody	Positive
CMV quantitative PCR	694963 IU/mL
HIV-1 serology (combination antigen/antibody immunoassay)	Positive
HIV-1 RNA load	323141 copies/mL
Absolute CD4 count	0 cel/μL (ref 256 – 1594 cells/UL)

Computed tomography (CT) brain was done, which showed no acute insult, followed by lumbar puncture (LP), which revealed negative results for diverse bacterial, viral infections, including JC virus (John Cunningham virus) and CMV PCR, and fungal infections including cryptococcus antigen. Magnetic resonance imaging (MRI) brain was done, which showed central atrophy of the brain parenchyma, small focal area of encephalomalacia, and gliosis in the left frontal pole, likely from an old infarct. MRI spine was done and revealed mild diffuse atrophy of the spinal cord, which is suggestive of HIV myelopathy. A nerve conduction study was performed, which showed severe sensorimotor axonal neuropathy in the lower limbs.

**Figure 1:**
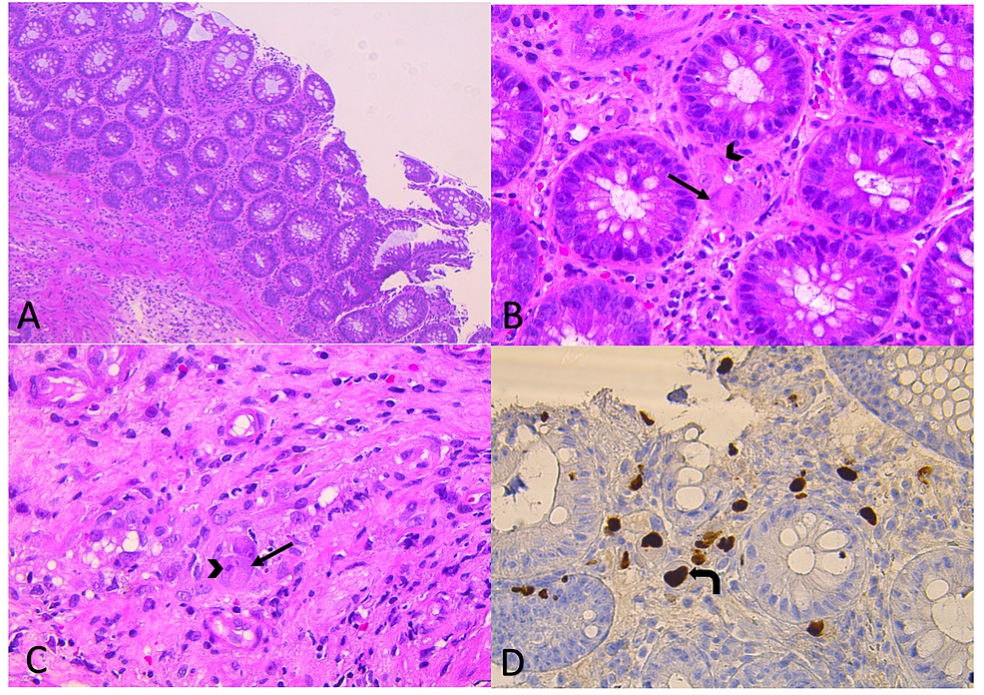
Tissue biopsy obtained from the colon Colonic mucosa with maintained crypt architecture and increased number of acute inflammatory cells, including eosinophils in the lamina propria. (A, H&E x 10): A viral cytopathic effect was noted in both the stromal cells; (B, H&E x40); and endothelial cells (C, H&E x40) The infected cells show cellular enlargement with an enlarged nucleus that shows prominent basophilic intranuclear inclusion surrounded by a clear halo, giving the cell an “Owl eye” appearance (arrowhead), The cytoplasm show also basophilic coarse granules, which constitute viral inclusion bodies (arrow). The immunohistochemical stain for cytomegalovirus was positive, as the virus-infected cells were staining brown (curved arrow, D, Immunohistochemistry x 40). H&E: Hematoxylin and Eosin

Given the patient's bloody diarrhea with positive CMV (viral load of 694963 IU/mL) and HIV, the gastroenterology team was consulted, and they decided to proceed with colonoscopy and biopsy. Colonoscopy was done and revealed multiple colonic ulcers with mild loss of vascularity on the left side colon. A biopsy was taken, which came positive for CMV colitis (Figure 1).

The ophthalmology team was consulted to rule out CMV retinitis. Ophthalmologic examination revealed cotton wall spots in the inferotemporal quadrant with a healthy optic nerve in the right eye while the left eye revealed retinitis in the inferonasal quadrant with overlying hemorrhage in addition to cotton wall spots in the superior temporal quadrant. The optic nerve was healthy in the left eye. The mentioned features are suggestive of CMV retinitis.

The patient was started on ganciclovir 5 mg/kg IV twice per day. He completed 12 days of treatment with marked improvement in his symptoms, including diarrhea. He was then discharged on valganciclovir 900 mg twice per day, to be followed in the clinic. Additionally, after five days of starting CMV treatment, the patient was started on antiretroviral treatment with Bktarvy (Bictegravir 50 mg, Emtricitabine 200 mg, Tenofovir 25 mg) one tablet a day; also, trimethoprim-sulfamethoxazole one double-strength tablet orally daily was started for Pneumocystis pneumonia (PCP). The patient was followed up in the ophthalmology clinic after three weeks from hospital discharge; a fundoscopic examination was done, which revealed resolution of CMV retinitis. He was also followed up in the infectious diseases clinic, which revealed improvement in overall clinical status, including gastrointestinal and neurological symptoms.

## Discussion

The simultaneous occurrence of CMV colitis and retinitis as the initial presentation of HIV infection is extremely rare; hence, our case is unique in its presentation. In some cases, CMV colitis and retinitis were presented as the only manifestation of HIV infection [3-5]. The risk of CMV retinitis and colitis is the highest when CD4 counts are below 50 cells/mm3 and is rare with CD4+ counts of more than 100 cells/mm3 [8]. Timely diagnosis of CMV infections is therefore very important in patients with acquired immunodeficiency syndrome (AIDS). Further complications of CMV infections can be prevented by early diagnosis and treatment [8].

Retinitis is the most common manifestation of CMV disease in HIV-positive patients [1]. The virus spreads to the retina by hematogenous spread; therefore, patients with serum CMV load are at an increased risk of developing CMV retinitis [9]. The progression of retinitis without specific treatment causes irreversible visual loss. The main symptoms of retinitis are a decrease in visual acuity (67%), floaters (49%), and flashing lights (16%); in addition, ocular irritation may present. CMV retinitis happens to be a very slow and progressive disease in patients with advanced-stage HIV [5-10]. The disease is diagnosed by fundoscopy unlike other systemic manifestations of CMV, which require confirmation of the diagnosis by biopsy taken from the affected site. Characteristic findings on fundoscopic examination include dense retinal whitening, hemorrhage, and a typical ‘brush-fire’ retinitis pattern that distributes along blood vessels with small white satellite lesions at the border [5]. These findings may be found in one eye first (as in our case, the patient’s left eye was affected) and then in the other eye. Retinal detachment may also occur with CMV retinitis. Proper management is essential for controlling the disease progression, prevention of relapses, and contralateral eye involvement.

Immediate administration of intravitreal ganciclovir or foscarnet in addition to systemic therapy for CMV is recommended for patients with sight-threatening lesions. Subsequent intravitreal treatment may be stopped if initial systemic therapy started within 24 hours of intravitreal treatment. However, patients without a sight-threatening infection can be treated with systemic therapy alone. The preferred systemic therapy is oral valganciclovir [11]. There is no consensus on when to start ART in the setting of CMV retinitis and HIV infection; however, in a high-income setting, ART is suggested to start approximately two weeks after starting treatment for CMV [12].

CMV colitis is also observed here as the initial manifestation of HIV. In a study, it was observed that CMV colitis was the index diagnosis of HIV in 11 out of 44 patients [8]. Hence, CMV colitis is a strong indication of the possibility of HIV. CMV colitis can be present early in HIV and often with nonspecific symptoms such as fever, intermittent diarrhea, weight loss, and haematochezia [13]. Notably, colonoscopy can be normal in CMV patients, requiring full colonoscopy with multiple biopsies for accurate diagnosis [14]. In our case, the patient presented with adnominal pain, diarrhea, fever, and weight loss.

Active CMV colitis is normally identified by endoscopic CMV discovery in colonic tissue, histological tests, including hematoxylin and eosin (H&E) and immunohistochemical (IHC) staining, and/or tissue PCR. H&E staining shows the conventional “owl’s eye” feature; the nuclei of cytomegalic cells containing CMV inclusion bodies are surrounded by clear cytoplasm [15-16]. Given our patient's bloody diarrhea with positive CMV and HIV, the gastroenterology team was consulted, and they decided to proceed with colonoscopy and biopsy [16]. Colonoscopy was done and revealed multiple colonic ulcers with mild loss of vascularity on the left side colon; biopsy was taken, which showed staining of colonic mucosal tissue, confirming the diagnosis of CMV colitis.

Treatment of CMV colitis includes the use of antiviral therapy with ganciclovir or valganciclovir [17]. ART therapy should be started once the patient can tolerate oral medications in the setting of CMV colitis [17]. Our patient had concurrent diagnoses of CMV colitis and retinitis. He was started on ART after five days from starting treatment for CMV, he improved with no evidence of immune reconstitution inflammatory syndrome (IRIS).

## Conclusions

The importance of our case, as it is extremely rare since CMV colitis and retinitis occurred simultaneously in a patient who was newly diagnosed with HIV with a zero CD4 count. We encourage clinicians to strictly refer newly diagnosed HIV patients with evidence of CMV disease, such as CMV colitis, to the ophthalmology team for a proper examination to rule out CMV retinitis because management will be different in those patients. Multidisciplinary teams, including infectious diseases, gastroenterology, ophthalmology, and pathology, should cooperate to facilitate accurate and fast diagnosis. Further complications can be prevented by early diagnosis and treatment.

## References

[1] Heinemann M-H (1992). Characteristics of cytomegalovirus retinitis in patients with acquired immunodeficiency syndrome. Am J Med.

[2] Holland GN, Vaudaux JD, Jeng SM (2008). Characteristics of untreated AIDS-related cytomegalovirus retinitis. I. Findings before the era of highly active antiretroviral therapy (1988 to 1994). Am J Ophthalmol.

[3] Paparone PP, Paparone PA (2012). Cytomegalovirus colitis in a human immunodeficiency virus-positive patient with a normal CD4 count. Am J Med Sci.

[4] Roskell DE, Hyde GM, Campbell AP, Jewell DP, Gray W (1995). HIV associated cytomegalovirus colitis as a mimic of inflammatory bowel disease. Gut.

[5] Hassan-Moosa R, Chinappa T, Jeena L, Visser L, Naidoo K (2017). Cytomegalovirus retinitis and HIV: case reviews from KwaZulu-Natal Province, South Africa. S Afr Med J.

[6] Leenasirimakul P, Liu Y, Jirawison C (2016). Risk factors for CMV retinitis among individuals with HIV and low CD4 count in northern Thailand: importance of access to healthcare. Br J Ophthalmol.

[7] Johnstone J, Boyington CR, Miedzinski LJ, Chiu I (2009). Cytomegalovirus colitis in an HIV-positive woman with a relatively preserved CD4 count. Can J Infect Dis Med Microbiol.

[8] Dieterich DT, Rahmin M (1991). Cytomegalovirus colitis in AIDS: presentation in 44 patients and a review of the literature. J Acquir Immune Defic Syndr (1988).

[9] Ferreira Tátá C, Ramires T, Piteira M, Matono R, Guz C (2021). Cytomegalovirus retinitis as a sole manifestation of HIV infection. Cureus.

[10] Au Eong KG, Beatty S, Charles SJ (1999). Cytomegalovirus retinitis in patients with acquired immune deficiency syndrome. Postgrad Med J.

[11] Holland GN (2008). AIDS and ophthalmology: the first quarter century. Am J Ophthalmol.

[12] (2022). Panel on Opportunistic Infections in HIV-Infected Adults and Adolescents. Guidelines for the prevention and treatment of opportunistic infections in adults and adolescents with HIV. Recommendations from the Centers for Disease Control and Prevention, the National Institutes of Health, and the HIV Medicine Association of the Infectious Diseases Society of America. https://clinicalinfo.hiv.gov/sites/default/files/guidelines/documents/Adult_OI.pdf.

[13] Adeiza MA, Habib AG (2019). A cross-sectional study of cytomegalovirus retinitis in HIV-1 infected adults in Nigeria. Niger J Clin Pract.

[14] Yoshida S, Mori N, Honda M (2019). Cytomegalovirus colitis in a patient with HIV infection shortly after initiation of antiretroviral therapy. IDCases.

[15] Yerushalmy-Feler A, Padlipsky J, Cohen S (2019). Diagnosis and management of CMV colitis. Curr Infect Dis Rep.

[16] Alukal J, Asif M, Mundada R, McNamee WB (2018). Recurrent cytomegalovirus colitis: a rare case of immune reconstitution inflammatory syndrome. BMJ Case Rep.

[17] Azer SA, Limaiem F (2021). Cytomegalovirus Colitis. https://www.ncbi.nlm.nih.gov/books/NBK542231/.

